# Voriconazole-associated adverse drug reactions in Chinese patients: incidence, clinical features and the predictive value of ALBI score for hepatotoxicity

**DOI:** 10.3389/fphar.2026.1839608

**Published:** 2026-07-10

**Authors:** Lin Hu, Yuan Su, Xi Tang, Yanfei Li, Ju Hou

**Affiliations:** 1 Department of Pharmacy, The Affiliated Changsha Hospital of Xiangya School of Medicine, Central South University, Changsha, Hunan, China; 2 Department of Pharmacy, The First Hospital of Changsha, Changsha, Hunan, China

**Keywords:** adverse drug reactions, risk factors, safety, therapeutic drug monitoring, voriconazole

## Abstract

**Objective:**

To analyze the incidence, types, and risk factors of adverse drug reactions (ADRs) associated with voriconazole (VRC).

**Methods:**

A prospective observational study was conducted at the First Hospital of Changsha from March 1, 2024, to December 31, 2025. Multivariate logistic regression analyses and receiver operating characteristic (ROC) curve analysis were performed to identify risk factors of ADRs.

**Results:**

A total of 370 patients were included in the analysis. ADRs occurred in 70 patients (18.9%), while 300 patients (81.1%) experienced no ADRs. The most common ADRs were hepatotoxicity (39/370, 10.5%), neurological disorders (20/370, 5.4%), and visual symptoms (18/370, 4.9%). Most ADRs developed within 4–7 days after treatment initiation (53.6%). Notably, 59 patients (84.3%) discontinued VRC due to ADRs. Among patients with hepatotoxicity, the median trough concentration (*C*_trough_) was 4.40 mg/L (range, 1.43–10.01 mg/L), which was significantly higher than that in the non-ADR group (3.29 mg/L; range, 0.64–12.80 mg/L; *P* = 0.016). No significant association was found between VRC *C*_trough_ and either neurological disorders or visual symptoms. However, patients who experienced visual symptoms received a significantly higher VRC dose (8.00 mg/kg/day; range, 5.00–12.00 mg/kg/day) compared with those without visual symptoms (7.02 mg/kg/day; range, 1.19–13.33 mg/kg/day) (*P* = 0.029). Multivariate logistic regression analysis identified VRC *C*_trough_ and albumin–bilirubin (ALBI) score as significant predictors of hepatotoxicity (*P* = 0.033 and *P* = 0.036, respectively). ROC analysis further indicated that patients with an ALBI score > −2.095 were at increased risk of developing hepatotoxicity.

**Conclusion:**

Early monitoring of VRC *C*_trough_ is essential. In addition to trough levels, the ALBI score demonstrated good predictive value for hepatotoxicity and may represent a promising new approach for predicting hepatotoxicity.

## Introduction

Voriconazole (VRC), a broad-spectrum triazole antifungal agent, is widely recommended as the first-line therapy for invasive aspergillosis (Patterson et al., 2016). Despite its well-established efficacy, concerns regarding toxicity have become increasingly prominent and pose substantial challenges in clinical practice. Previous studies have established a concentration-dependent relationship between VRC exposure and adverse drug reactions (ADRs) (Dolton et al., 2012). The safety of VRC remains a major clinical concern, as ADRs may lead to treatment interruption and consequently compromise antifungal effectiveness. In a previous study of patients with pre-existing liver dysfunction, we found that VRC was associated with multiple ADRs (Hu et al., 2024). However, that analysis was confined to a specific high-risk subpopulation. The present study extends these findings by evaluating the broader spectrum of VRC-related ADRs in a general adult inpatient cohort.

VRC is primarily metabolized in the liver by cytochrome P450 (CYP) isoenzyme 2C19. Previous studies have demonstrated that VRC trough concentration (*C*_trough_) in CYP2C19 poor metabolizers (PMs) were markedly higher compared with those in intermediate metabolizers (IMs) and normal metabolizers (NMs) (Du et al., 2024). In addition, several studies have reported that PMs exhibited an increased risk of hepatotoxicity compared with NMs (Adachi et al., 2025). The prevalence of CYP2C19 PMs is significantly higher in Asian populations than in European and American populations (PharmGKB.org, 2025). This may lead to differences in the incidence of ADRs between Asian and non-Asian populations. A consensus guideline from the Japanese Society of Chemotherapy noted that Asian populations may be at higher risk of supratherapeutic VRC exposure and related ADRs than non-Asian populations, and therefore recommended a lower upper trough target for Asians (<4.0 mg/L) than for non-Asians (<5.5 mg/L) (Takesue et al., 2022).

The albumin-bilirubin (ALBI) score is an objective measure of liver functional reserve calculated from serum albumin (ALB) and total bilirubin (TBil). Unlike the Child-Pugh score, it excludes subjective components and allows continuous risk stratification (Johnson et al., 2015). Recent evidence suggests the ALBI score may predict VRC-induced hepatotoxicity (Asai et al., 2025). Nevertheless, its predictive value has not been prospectively validated in a Chinese cohort, and an optimal cutoff for risk stratification has not been established.

To address these gaps, we conducted a prospective observational study in a general hospitalized adult cohort with three specific objectives: (i) to characterize the full spectrum of VRC-related ADRs in a general Chinese inpatient population; (ii) to analyze the potential factors associated with VRC-related ADRs; and (iii) to prospectively validate the ALBI score as a predictor of VRC-induced hepatotoxicity and determine an optimal cutoff.

## Patients and methods

### Study population and study design

A non-interventional, prospective observational study was conducted from March 1, 2024, to December 31, 2025, at the First Hospital of Changsha, a large tertiary care center with 2,000 beds. This study primarily evaluated the incidence and spectrum of VRC-related ADRs among hospitalized Chinese patients. ADRs were closely monitored and comprehensively documented in hospitalized patients.

The inclusion criteria were: (i) age ≥18 years; (ii) receipt of VRC therapy with at least one steady-state *C*_trough_ measurement; (iii) a VRC treatment duration of ≥3 days; and (iv) patients who received VRC for either treatment or prophylaxis. The exclusion criteria were: (i) VRC *C*_trough_ not at steady state; (ii) absence of liver function monitoring before and after VRC administration; and (iii) concomitant use of other strongly hepatotoxic medications (e.g., chemotherapy or hepatotoxic antibiotics) during the hepatotoxicity assessment period.

Patients were monitored for ADRs from the initiation of VRC therapy until hospital discharge. No missing data were present for the key variables analyzed in this study. As this was an exploratory prospective observational study, no formal sample size calculation was performed. All eligible patients treated with VRC during the study period were consecutively enrolled.

## Ethics approval

This study was conducted in strict accordance with the Declaration of Helsinki and approved by the Ethics Committee of the First Hospital of Changsha (approval number: 2024008). Written informed consent was obtained from all participants. All patient information was coded to ensure that no identifiable data were disclosed.

### Data collection

Patient medical records were collected, including demographic and clinical data such as age, body weight, sex, underlying diseases, concomitant medications, VRC *C*_trough_, VRC dosage, administration route, treatment duration, and laboratory parameters, including C-reactive protein (CRP), ALB, TBil, direct bilirubin (DBil), alanine aminotransferase (ALT), aspartate aminotransferase (AST), alkaline phosphatase (ALP) and serum creatinine (Scr). The degree of inflammation was evaluated using CRP levels. Patients were stratified into three CRP groups according to inflammation severity: <40 mg/L (mild inflammation), 40–100 mg/L (moderate inflammation), and >100 mg/L (severe inflammation) (Hu et al., 2024). Patients were also categorized by age (<60 years and ≥60 years) (Cheng et al., 2020), and by ALB level (<30 g/L and ≥30 g/L) (Sánchez-Rodríguez et al., 2018). For patients who experienced ADRs, detailed information was recorded, including time of onset, clinical manifestations, causality assessment, prognosis, and VRC dose adjustments. Data were independently collected by two researchers, with discrepancies resolved through consultation with a third researcher.

### Criteria for assessing the causality and severity of ADRs

Assessment of hepatotoxicity: Roussel Uclaf Causality Assessment Method (RUCAM) (Danan et al., 2015) was used to evaluate the causality between VRC and hepatotoxicity. A score >8 indicates a highly probable association, 6–8 probable, 3–5 possible, one to two unlikely, and ≤0 excluded. VRC-associated hepatotoxicity was defined as a RUCAM score ≥3. The severity of hepatotoxicity was assessed using the Drug-Induced Liver Injury (DILI) severity grading scale (Brennan et al., 2021). This classification is based on serum biochemical parameters—ALT, ALP, their ratios to the upper limit of normal (ULN), and TBil. The specific grading criteria are as follows:

Grade 1: ALT ≥ 5 × ULN or ALP ≥ 2 × ULN, and TBil < 2 × ULN.

Grade 2: ALT ≥ 5 × ULN or ALP ≥ 2 × ULN, and TBil ≥ 2 × ULN, or symptomatic hepatitis.

Grade 3: ALT ≥ 5 × ULN or ALP ≥ 2 × ULN, and TBil ≥ 2 × ULN, or symptomatic hepatitis accompanied by any of the following: (i) INR ≥ 1.5; (ii) ascites and/or hepatic encephalopathy with a disease course < 26 weeks and no cirrhosis; (iii) other organ failure attributable to DILI.

Grade 4: Acute liver failure, death, or liver transplantation due to DILI.

Assessment of other ADRs: The causality of other ADRs related to VRC was assessed using the WHO-UMC system (Pandit et al., 2024), categorized as certain, probable, possible, unlikely, conditional, or unassessable. A VRC-attributable ADR was defined as an event with a causality assessment of possible or higher. The severity of these ADRs was graded according to the Common Terminology Criteria for Adverse Events (CTCAE), version 5.0 (CTCAE, 2024). Central nervous system (CNS) toxicity included visual symptoms such as blurred vision, photophobia, color vision changes (encompassing green vision, blue vision, xanthopsia, altered color perception, and color blindness), photopsia, reduced visual acuity, vitreous floaters, as well as neurological disorders including depression, hallucinations, dizziness, anxiety, insomnia, and depressed level of consciousness. Elevated creatinine was classified as at least grade 1 (estimated glomerular filtration rate [eGFR] or creatinine clearance [CrCl] <60 mL/min/1.73 m^2^, or proteinuria ≥2+; urine protein-to-creatinine ratio >0.5) or above.

During the study period, patients underwent regular monitoring of liver function and were actively assessed for visual symptoms and neurological disorders. Once an ADR was identified, its severity was classified according to the criteria described above, and this classification served as the basis for subsequent statistical analyses.

### VRC administration and monitoring of VRC *C*_trough_

According to the VRC prescribing information, the recommended dosing regimen includes an intravenous loading dose of 6 mg/kg every 12 h (q12h), followed by a maintenance dose of 4 mg/kg q12h, or an oral loading dose of 400 mg q12h, followed by a maintenance dose of 200 mg q12h. Dose adjustments were guided by the Chinese Pharmacological Society guideline (Chen et al., 2018). For *C*_trough_ <0.5 mg/L, the daily dose was increased by 50%. For *C*_trough_ of 5.0–10 mg/L without ≥ grade 2 adverse events, the dose was reduced by 20%. For *C*_trough_ >10 mg/L, or in the presence of grade 2 adverse events, one dose was withheld and the maintenance dose was reduced by 50%. According to the same guideline, when a loading dose is administered on the first day, blood sampling for steady-state *C*_trough_ measurement is performed on the third day of therapy. If no loading dose is given, sampling is conducted on the fourth day. Once steady-state concentrations are achieved, 2 mL of venous blood is collected within 30 min prior to the next scheduled dose to determine the VRC *C*_trough_. VRC *C*_trough_ levels were quantified using a liquid chromatography–tandem mass spectrometry (LC–MS/MS) method, as described in our previous study (Hu et al., 2024). In summary, the linear calibration range was 0.10–20.00 mg/L. The intraday and interday precision values were 2.23%–3.18% and 3.73%–4.57%, respectively. The extraction recovery rate ranged from 104.24% to 109.37%, and the relative standard deviation (RSD) of the normalized matrix effect factor for the internal standard was 6.12%–11.63%. The therapeutic range for VRC *C*_trough_ is 1.0–5.5 mg/L.

### Statistical analysis

Categorical variables are presented as counts and percentages (n [%]), while continuous variables, which did not follow a normal distribution, are expressed as medians with ranges. Spearman’s rank correlation coefficient was used to assess associations between continuous variables. Comparisons of categorical variables were conducted using the chi-square test, and comparisons of continuous variables were performed using the nonparametric Mann-Whitney U test. Multivariate logistic regression models were used to identify potential risk factors for ADRs, including sex, route of administration, VRC dosage, *C*_trough_, age, degree of inflammation, ALBI score, and concomitant medications. Cut-off values were determined as the points corresponding to the maximum area under the curve (AUC) on the receiver operating characteristic (ROC) curve. The ALBI score was calculated using ALB and TBil values measured at baseline. The ALBI score was calculated using the formula:

ALBI = (0.66 × log_10_[TBIL, μmol/L]) − (0.085 × ALB [g/L]).

ALBI grades were categorized as follows: grade 1 (≤ −2.60), grade 2 (−2.59 to −1.39), and grade 3 (> −1.39), with higher scores indicating poorer liver function (Adachi et al., 2025). All statistical analyses were performed using SPSS software (version 25.0), and a two-sided *P*-value < 0.05 was considered statistically significant.

## Results

### Patient characteristics

**FIGURE 1:**
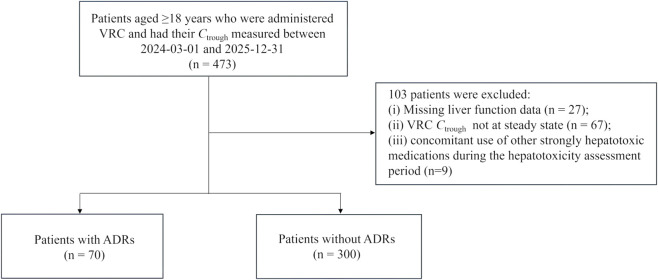
Flow diagram of patients screening. VRC, voriconazole. *C*_trough_, trough concentration. ADRs, adverse drug reactions.

Of the 473 patients initially treated with VRC, 103 were excluded due to missing liver function data (n = 27), VRC *C*_trough_ not at steady state (n = 67), and concomitant use of other strongly hepatotoxic medications during the hepatotoxicity assessment period (n = 9). The remaining 370 patients comprised the study cohort, including 70 in the ADR group and 300 in the non-ADR group. The patient screening process is presented in Figure 1.

In the ADR group, the median age was 69 years (range, 24–100 years), with 68.6% (48/70) aged ≥ 60 years. Males accounted for 72.9% (51/70). Malignancy was the most common underlying disease (28.6%). The predominant route of administration was intravenous (77.1%). The most common medication administered concomitantly was proton pump inhibitors (PPIs) (20.0%). The median CRP level was 36.67 mg/L (range, 0.61–268.94 mg/L), and 32.9% of patients had ALB levels < 30 g/L. In the non-ADR group, the median age was 69 years (range, 18–100 years), with 71.3% (214/300) aged ≥ 60 years. Males accounted for 63.3% (190/300). Malignancy was present in 29.7% of patients. The majority of patients (83.7%) received intravenous administration, with 18.3% co-administered PPIs. The median CRP level was 49.4 mg/L (range, 0.23–341.50 mg/L), and 34.3% of patients had ALB levels < 30 g/L. In the ADR group, VRC was used for therapeutic, empirical, and prophylactic indications in 35 (50.0%), 21 (30.0%), and 14 (20.0%) patients, respectively. In the non-ADR group, the corresponding proportions were 161 (53.7%), 89 (29.7%), and 50 (16.7%). Patient characteristics are summarized in Table 1.

**TABLE 1:** Patient characteristics.

Characteristics	All patients (*n* = 370)	Grouped by the occurrence of ADRs		
		ADR group (*n* = 70)	Non-ADR group (*n* = 300)	*P* value
Sex, n (%) Male Female	241 (65.1) 129 (34.9)	51 (72.9) 19 (27.1)	190 (63.3) 110 (36.7)	0.132
Age (year), median (range) < 60 years, n (%) ≥ 60 years, n (%)	69 (18–100) 108 (29.2) 262 (70.8)	69 (24–100) 22 (31.4) 48 (68.6)	69 (18–100) 86 (28.7) 214 (71.3)	0.765 0.647
Body weight (kg), median (range)	56.25 (30–110)	55 (40–100)	57 (30–110)	0.846
Underlying condition, n (%) Malignancy COPD AIDS Liver cirrhosis Chronic renal insufficiency Others	109 (29.5) 88 (23.8) 32 (8.6) 34 (9.2) 13 (3.5) 94 (25.4)	20 (28.6) 11 (15.7) 9 (12.9) 4 (5.7) 4 (5.7) 22 (31.4)	89 (29.7) 77 (25.7) 23 (7.7) 30 (10.0) 9 (3.0) 72 (24.0)	0.856 0.078 0.164 0.264 0.453 0.199
IFD diagnosis, n (%) Proven Probable Possible	160 (43.2) 118 (31.9) 92 (24.9)	26 (37.1) 24 (34.3) 20 (28.6)	134 (44.7) 94 (31.3) 72 (24.0)	0.253 0.633 0.426
VRC treatment indication, n (%) Therapeutic Empirical Prophylactic	196 (53.0) 110 (29.7) 64 (17.3)	35 (50.0) 21 (30.0) 14 (20.0)	161 (53.7) 89 (29.7) 50 (16.7)	0.580 0.956 0.507
VRC treatment duration (days), median (range)	10 (3–54)	8 (3–54)	10 (3–40)	0.096
VRC *C*_trough_ (mg/L), median (range)	3.31 (0.64–12.80)	3.69 (0.88–10.01)	3.29 (0.64–12.80)	0.316
VRC maintenance dose (mg/kg/day), median (range)	7.10 (1.19–13.33)	7.27 (2.35–12.00)	7.27 (2.35–12.00)	0.514
Administration route, n (%) Oral Intravenous	65 (17.6) 305 (82.4)	16 (22.9) 54 (77.1)	49 (16.3) 251 (83.7)	0.197
Combined use of PPIs, n (%) yes no	69 (18.6) 301 (81.4)	14 (20.0) 56 (80.0)	55 (18.3) 245 (81.7)	0.747
Combined use of glucocorticoid, n (%) yes no	58 (15.7) 312 (84.3)	13 (18.6) 57 (81.4)	45 (15.0) 255 (85.0)	0.459
CRP (mg/L), median (range) < 40 mg/L, n (%) 40–100 mg/L, n (%) > 100 mg/L, n (%)	47.32 (0.23–341.50) 176 (47.6) 104 (28.1) 90 (24.3)	36.67 (0.61–268.94) 37 (52.9) 16 (22.9) 17 (24.3)	49.4 (0.23–341.50) 139 (46.3) 88 (29.3) 73 (24.3)	0.465 0.325 0.278 0.993
ALBI grade, n (%) grade 1 grade 2 grade 3	58 (15.7) 277 (74.9) 35 (9.5)	4 (10.3) 31 (79.5) 4 (10.3)	54 (16.3) 246 (74.3) 31 (9.4)	0.325 0.482 0.857
ALB (g/L), median (range) < 30 g/L, n (%) ≥ 30 g/L, n (%)	31.60 (13.10–71.30) 126 (34.1) 244 (65.9)	31.40 (18.70–45.30) 23 (32.9) 47 (67.1)	31.65 (13.10–71.30) 103 (34.3) 197 (65.7)	0.904 0.814

ADRs, adverse drug reactions; COPD, chronic obstructive pulmonary disease; AIDS, acquired immune deficiency syndrome; IFD, invasive fungal disease; VRC, voriconazole; PPIs, proton pump inhibitors; CRP, C-reactive protein. ALB, albumin; kg, kilogram; ALBI, albumin-bilirubin.

### Characteristics of VRC-related ADRs

The overall incidence of ADRs was 18.9% (70/370). The three most frequent ADRs were hepatotoxicity (39 patients, 10.5%), neurological disorders (20 patients, 5.4%), and visual symptoms (18 patients, 4.9%). Other ADRs included rash (3 patients, 0.8%), elevated creatinine (1 patient, 0.3%), leukopenia (1 patient, 0.3%), chest pain (1 patient, 0.3%), and nausea (1 patient, 0.3%). Fourteen patients experienced two types of ADRs simultaneously. The relationship between ADRs and VRC was classified as probable in 56 patients (80.0%) and possible in 14 patients (20.0%). In the ADR group, 90.0% of patients required dose adjustments, with VRC discontinued in 59 patients (84.3%) and dose reduction implemented in 4 patients (5.7%). The prognosis of ADRs in these patients were favorable, with 82.9% cured and 17.1% improved. Detailed characteristics of VRC-related ADRs are summarized in Table 2.

**TABLE 2:** Details of ADRs related to VRC during the treatment.

Characteristics	Different types of ADRs			
	Hepatotoxicity	Neurological disorders	Visual symptoms	Other ADRs
No. of samples (incidence)	39 (10.5%)	20 (5.4%)	18 (4.9%)	7 (1.9%)
Clinical manifestations of ADRs	ALT or AST increased, *n* = 34; hyperbilirubinemia, *n* = 5	Depressed level of consciousness, *n* = 10; hallucination, *n* = 6; dizzy, *n* = 4	Blurred vision, *n* = 14; reduced visual acuity, *n* = 2; photopsia, *n* = 1; color vision changes, *n* = 1	Rash, *n* = 3; creatinine increased, *n* = 1; leukopenia, *n* = 1; chest pain, *n* = 1; nausea, *n* = 1
Severity of ADRs, n (%)	DILI grade 1: 24 (61.5); grade 2: 7 (17.9); grade 3: 7 (17.9); grade 4: 1 (2.6)	CTCAE grade 1: 18 (90.0); grade 3: 2 (10.0)	CTCAE grade 1: 18 (100.0)	CTCAE grade 1: 5 (71.4); grade 2: 2 (28.6)
Casualty assessment, n (%) Probable Possible	33 (84.6) 6 (15.4)	19 (95.0) 1 (5.0)	13 (72.2) 5 (27.8)	3 (42.9) 4 (57.1)
Prognosis, n (%) Cure Improvement	29 (74.4) 10 (25.6)	18 (90.0) 2 (10.0)	17 (94.4) 1 (5.6)	4 (57.1) 3 (42.9)
Age group, n (%) < 60 years ≥ 60 years	10 (25.6) 29 (74.4)	7 (35.0) 13 (65.0)	5 (27.8) 13 (72.2)	3 (42.9) 4 (57.1)
Administration route, n (%) oral Intravenous	8 (20.5) 31 (79.5)	7 (35.0) 13 (65.0)	2 (11.1) 16 (88.9)	3 (42.9) 4 (57.1)
Dose changes after ADRs occurrence, n (%) discontinuation dose reduction	34 (87.2) 3 (7.7)	18 (90.0) 1 (5.0)	13 (72.2) 1 (5.6)	7 (100.0) 0
CRP concentration, n (%) < 40 mg/L 40–100 mg/L > 100 mg/L	19 (48.7) 11 (28.2) 9 (23.1)	10 (50.0) 2 (10.0) 8 (40.0)	13 (72.2) 3 (16.7) 2 (11.1)	6 (85.7) 0 1 (14.3)

ADRs, adverse drug reactions; VRC, voriconazole; CRP, C-reactive protein; DILI, drug-induced liver injury; CTCAE, Common Terminology Criteria for Adverse Events; ALT, alanine aminotransferase; AST, aspartate aminotransferase.

Regarding the time of onset, most ADRs occurred within 4–7 days after VRC administration (53.6%). Specifically, hepatotoxicity was most frequently observed within 4–7 days (64.1%), neurological disorders within 2–7 days (70.0%), and visual symptoms within 4–7 days (66.7%). Notably, with the exception of one case of rash that occurred after 14 days, all ADRs developed within the first 14 days of VRC therapy. The onset times of ADRs following VRC administration are presented in Table 3.

**TABLE 3:** The time from VRC administration to the onset of the ADRs.

ADRs	Time interval (days), n (%)				
	≤1	2–3	4–7	8–14	>14
Hepatotoxicity	0	9 (23.1)	25 (64.1)	5 (12.8)	0
Neurological disorders	2 (10.0)	7 (35.0)	7 (35.0)	4 (20.0)	0
Visual symptoms	1 (5.6)	4 (22.2)	12 (66.7)	1 (5.6)	0
Rash	1 (33.3)	1 (33.3)	0	0	1 (33.3)
Elevated creatinine	0	1 (100.0)	0	0	0
Leukopenia	0	0	0	1 (100.0)	0
Chest tightness	0	1 (100.0)	0	0	0
Gastrointestinal reactions	0	0	1 (100.0)	0	0
Total	4 (4.8)	23 (27.4)	45 (53.6)	11 (13.1)	1 (1.2)

VRC, voriconazole; ADRs, adverse drug reactions.

A significant positive correlation was observed between VRC *C*_trough_ and ALBI score (r = 0.200, *P* < 0.001), and patients who developed hepatotoxicity had significantly higher ALBI scores than those without hepatotoxicity (*P* = 0.003). Among the enrolled patients, 58 were classified as ALBI grade 1, 277 as ALBI grade 2, and 35 as ALBI grade 3. The incidence of hepatotoxicity was 6.9% (4/58), 11.2% (31/277), and 11.4% (4/35) in ALBI grades 1, 2, and 3, respectively, with no significant difference among groups (*P* = 0.616).

### Correlation of ADRs with VRC *C*_trough_ and dosage

**FIGURE 2:**
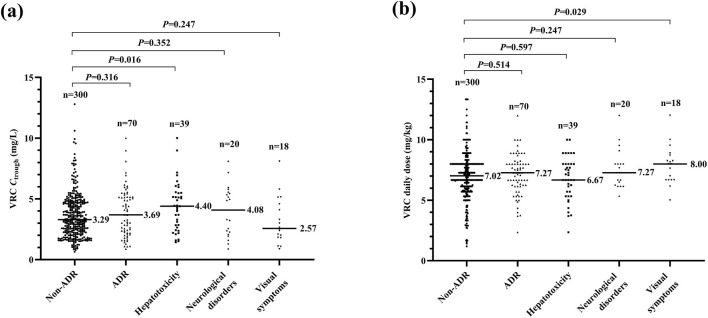
The distribution of VRC *C*_trough_ **(a)** and daily dose **(b)** among the non-ADR, ADR, hepatotoxicity, neurological disorder, and visual symptom groups. Horizontal bars represent median VRC trough value for each group. *P*-values and number of patients are indicated above each plot.

The median VRC *C*_trough_ in the ADR group was 3.69 mg/L (range, 0.88–10.01 mg/L), which was not significantly different from that in the non-ADR group (3.29 mg/L; range, 0.64–12.80 mg/L; *P* = 0.316). Among patients with hepatotoxicity, the median *C*_trough_ was 4.40 mg/L (range, 1.43–10.01 mg/L), significantly higher than in the non-ADR group (*P* = 0.016). In contrast, patients with neurological disorders did not differ significantly from those in the non-ADR group (*P* = 0.352), and patients with visual symptoms also showed no significant difference compared with those in the non-ADR group (*P* = 0.247). No significant difference was observed in the daily VRC dose between patients who developed ADRs and those who did not (*P* = 0.514). Similarly, the daily VRC dose did not differ significantly between patients with and without hepatotoxicity (*P* = 0.597) or between those with and without neurological disorders (*P* = 0.247). However, patients who experienced visual symptoms received a significantly higher VRC dose (8.00 mg/kg/day; range, 5.00–12.00 mg/kg/day) compared with those without visual symptoms (7.02 mg/kg/dayg; range, 1.19–13.33 mg/kg/day) (*P* = 0.029). The distribution of VRC *C*_trough_ and daily dose among the non-ADR, ADR, hepatotoxicity, neurological disorder, and visual symptom groups is presented in Figures 2a,b.

### Risk factors for VRC-related ADRs by univariate and multivariate logistic regression analyses

Sex, route of administration, VRC dose, VRC *C*_trough_, age group, CRP group, ALBI score, and concomitant medications were evaluated as potential predictors of hepatotoxicity in univariate logistic regression analyses. In univariate analyses, both VRC *C*_trough_ and ALBI score were significantly associated with hepatotoxicity (*P* = 0.013 and *P* = 0.018, respectively). These associations remained significant in the multivariable logistic regression model (VRC *C*_trough_, *P* = 0.033; ALBI score, *P* = 0.036). Detailed results are presented in Table 4.

**TABLE 4:** Univariate and multivariate logistic analysis of risk factors for hepatotoxicity related to VRC.

Variables	Univariate analysis		Multivariate analysis	
	OR (95% CI)	*P* value	OR (95% CI)	*P* value
Female^a^	1.539 (0.727–3.256)	0.260	​	​
Oral administration^b^	0.937 (0.413–2.123)	0.875	​	​
VRC dose (mg/kg/day)	0.931 (0.782–1.109)	0.424	​	​
VRC *C*_trough_ (mg/L)	1.220 (1.042–1.429)	0.013	1.189 (1.014–1.394)	0.033
Age <60 years^c^	1.219 (0.575–2.587)	0.605	​	​
CRP group^d^ 40–100 mg/L >100 mg/L	0.997 (0.479–2.075) 0.923 (0.423–2.015)	0.994 0.840	​	​
ALBI score	2.042 (1.130–3.687)	0.018	1.917 (1.043–3.525)	0.036
Combined use of PPIs^e^	1.144 (0.504–2.598)	0.747	​	​
Combined use of glucocorticoids^f^	1.076 (0.362–3.201)	0.895	​	​

VRC, voriconazole; *C*_trough_, trough concentration; CRP, C-reactive protein. ALBI, albumin-bilirubin; PPIs, proton pump inhibitors; OR, odds ratio; CI, confidence interval.

^a^ Compared to male.

^b^ Compared to intravenous administration.

^c^ Compared with patients aged ≥60 years.

^d^ Compared to CRP <40 mg/L.

^e^ Compared to without combined use of PPIs.

^f^ Compared to without combined use of glucocorticoids.

No significant predictors were identified for neurological disorders. In univariate logistic regression analysis, only VRC dose was significantly associated with the occurrence of visual symptoms (*P* = 0.035). The corresponding analyses are provided in the Supplementary Material.

### ROC curve for predicting hepatotoxicity risk

**FIGURE 3:**
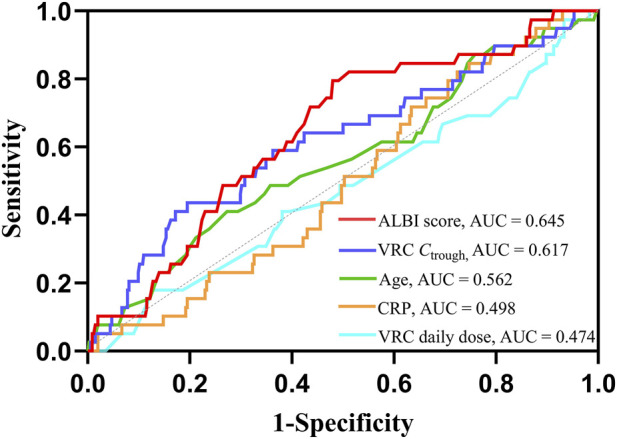
Receiver operating characteristic (ROC) curve of VRC *C*_trough_ to predict risk of hepatotoxicity. ALBI, albumin-bilirubin. VRC, voriconazole. *C*_trough_, trough concentration. CRP, C-reactive protein. AUC, area under the curve.

The ROC curve analysis demonstrated significant associations between hepatotoxicity and both ALBI score (AUC = 0.645; 95% CI: 0.559–0.731; cutoff = −2.095; *P* = 0.003) and VRC *C*_trough_ (AUC = 0.617; 95% CI: 0.518–0.716; cutoff = 4.89 mg/L; *P* = 0.016). In contrast, no significant associations were observed for age, CRP levels, or daily VRC dose (*P* = 0.204, *P* = 0.960, and *P* = 0.598, respectively). The ROC curve for predicting hepatotoxicity is shown in Figure 3.

## Discussion

This study summarized the incidence and types of VRC-related ADRs, evaluated their association with VRC *C*_trough_ and dosage, and identified potential risk factors. These findings provide important insights into the characteristics of VRC-related ADRs in the Chinese population. Recognizing ADRs such as VRC-associated hepatotoxicity, neurological disorders, and visual symptoms is crucial for ensuring the safety of VRC and maintaining treatment continuity.

In this study, the overall incidence of VRC-related ADRs was 18.9%, with hepatotoxicity being the most common (10.5%). A large retrospective analysis of 7,659 patients in China reported a similar incidence of VRC-induced liver injury (11.32%), supporting the consistency of our findings (Lou et al., 2025). Several studies have noted a higher incidence of VRC-associated hepatotoxicity in Asian populations. For instance, a real-world Chinese study reported elevated liver enzymes as the most frequent ADR, with an incidence of 42.9% (60/140). In that study, hepatotoxicity was associated with a *C*_trough_ >3.61 mg/L, with 66.7% of cases occurring within 7 days and 94.4% within 15 days of treatment initiation (Shen et al., 2022). A single-center study in Japan reported an incidence of 28.6% (12/42), with 91.7% (11/12) of hepatotoxicity cases occurring within 3 weeks of therapy, and the authors suggested maintaining *C*_trough_ <4.2 mg/L (Hirata et al., 2019). Another Japanese study similarly observed an incidence of 28.2% (11/39) and reported that persistently elevated *C*_trough_ markedly increased the risk of hepatotoxicity (Suzuki et al., 2013).

The incidence of CNS toxicity in this study was 10.3% (38/370), including 5.4% (20/370) for neurological disorders and 4.9% (18/370) for visual symptoms, with neither showing a significant association with VRC *C*_trough_. Clinical observations indicated that most neurological and visual adverse effects were mild, transient, and reversible, typically resolving spontaneously after dose reduction or drug discontinuation. A retrospective study from the United States reported a CNS toxicity incidence of 5% (16/320) and likewise found no correlation with VRC *C*_trough_ (Heo et al., 2017), consistent with our findings. In contrast, other studies in Chinese populations have documented higher rates of VRC-related CNS toxicity, including neurological disturbances and visual symptoms, occurring in up to 20.6% of patients (34/165), with a median onset of 6 days (range: 2–19 days). Moreover, elevated VRC *C*_trough_ has been identified as an independent risk factor for neurotoxicity, with significantly lower incidence observed when trough levels are maintained at ≤4.85 mg/L (Yang et al., 2022). Kato et al. (2024) similarly reported that higher VRC *C*_trough_ may increase the risk of visual symptoms.

An important finding of this study is that, with the exception of one rash case, all ADRs developed within the first 14 days of VRC therapy, and the majority (53.6%) occurred within 4–7 days. This temporal pattern suggests that intensive monitoring for hepatotoxicity, neurological disorders, and visual symptoms should be prioritized during the first 2 weeks of treatment. The single delayed rash case is consistent with the known later onset of drug-induced skin reactions. Although the lack of post-discharge follow-up may have missed late-onset events, these findings support early therapeutic drug monitoring (TDM) and close monitoring within the initial 1–2 weeks of VRC initiation.

In examining factors associated with VRC-related ADRs, hepatotoxicity has previously been linked to elevated VRC *C*_trough_ and higher ALBI grades (Asai et al., 2025; Cheng et al., 2024). Consistent with these findings, our study also identified a significant association between ALBI grade and the development of hepatotoxicity. Lou et al. (2025) reported that VRC-induced hepatotoxicity was associated with intravenous administration, elevated baseline urea, increased triglycerides (TG), elevated CRP, pre-existing liver disease, diabetes, and transplantation status. In contrast, our study did not observe a correlation between ADR occurrence and CRP levels. Yang et al. (2022) found that VRC *C*_trough_ significantly influenced the development of CNS toxicity, however, no such association was identified in our study.

Evidence regarding pharmacogenetic predictors of ADRs remains limited and inconclusive. Several studies have suggested that genetic polymorphisms in metabolic enzymes may modulate the risk of VRC-induced hepatotoxicity. For instance, the CYP51A1 variant (rs6465348) has been proposed to reduce hepatotoxicity, although further research is needed to clarify its clinical relevance (Takahashi et al., 2021). Numerous studies have confirmed that CYP2C19 polymorphisms significantly affect VRC *C*_trough_. Jansen et al. (Jansen et al., 2017) reported a case involving a 78-year-old patient with acute myeloid leukemia who carried the CYP2C19*2 allele and experienced musical hallucinations; the symptoms resolved after dose adjustment guided by pharmacogenetic testing. Given the high prevalence of the CYP2C19*2 allele in the Chinese population, its potential contribution to CNS toxicity warrants further investigation.

Although a systematic review and meta-analysis (Zhang et al., 2022) suggested that CYP2C19 PMs have a higher incidence of adverse events compared with NMs and IMs, other studies have not supported this association. Levin et al. (Levin et al., 2007) reported no significant differences in peak liver enzyme levels or maximum enzyme elevation across CYP2C19 genotypes. Similarly, Yang et al. found no meaningful impact of CYP2C19 polymorphisms on VRC-induced CNS toxicity, and Wu et al. (Wu et al., 2025) observed no association between CYP2C19 genotype and visual or neurological/psychiatric adverse events. Overall, these findings suggest that the predictive value of CYP2C19 genotyping for VRC-related ADRs may be limited.

CYP2C19 genotyping was not incorporated because it was not routinely available at our hospital during the study period and could not be uniformly funded for consecutive inpatients. Because our objective was not to evaluate pharmacogenetic testing, which remains unavailable in many clinical settings, we instead focused on validating a more broadly accessible predictor, the ALBI score, which can be derived from routine laboratory tests in any hospital. Nevertheless, the absence of CYP2C19 data limits our ability to fully disentangle the relationships among genotype, VRC *C*_trough_, ALBI score, and hepatotoxicity. Given the relatively high prevalence of CYP2C19 PM status in Asian populations, unmeasured genotype may have confounded the observed associations. Future prospective studies should incorporate both ALBI and CYP2C19 genotyping to clarify their independent and joint contributions to predicting VRC-related hepatotoxicity.

The mechanisms underlying VRC-induced hepatotoxicity remain incompletely understood, with oxidative stress and disrupted energy metabolism proposed as contributing pathways (Wu et al., 2019; Chiang et al., 2024). With respect to CNS toxicity, VRC can penetrate the blood–brain and blood–retina barriers (Henry et al., 2013), and emerging evidence suggests a potential role for dopaminergic signaling (Kato et al., 2024). In our study, baseline ALBI score showed modest discrimination for VRC-related hepatotoxicity (AUC = 0.645), with an optimal cutoff of > −2.095. These findings suggest that the ALBI score, an objective and readily available indicator of hepatic functional reserve, may be useful for pre-treatment risk stratification.

Notably, the cutoff of −2.095 falls within ALBI grade 2, underscoring heterogeneity within this category. Although ALBI grade may be useful for rapid screening, the continuous ALBI score (e.g., automatically calculated in electronic medical records) may provide more precise risk stratification when considering dose adjustment. Patients with ALBI scores > −2.095 may warrant closer monitoring and, when clinically appropriate, more conservative initial dosing, particularly in Asian populations with a higher prevalence of CYP2C19 PMs. Future prospective studies are needed to determine whether ALBI-guided dosing and monitoring strategies can reduce VRC-related hepatotoxicity without compromising antifungal efficacy.

Our ROC analysis identified a VRC *C*_trough_ cutoff of 4.89 mg/L for predicting hepatotoxicity (AUC, 0.617; 95% CI, 0.518–0.716). Although this threshold is higher than the 3.0 mg/L value reported in some meta-analyses (Jin et al., 2016), it is broadly consistent with the more conservative upper targets proposed for Asian populations (e.g., <4.0 mg/L) (Takesue et al., 2022). The discrepancy likely reflects heterogeneity in study populations and hepatotoxicity definitions, the limited discriminative ability of *C*_trough_ alone, and effect modification by host factors such as hepatic reserve as captured by the ALBI score.

Importantly, this finding should not be interpreted as indicating that trough levels above 4.89 mg/L are safe. Rather, it highlights substantial interindividual susceptibility and supports integrating exposure with host risk. In our cohort, ALBI performed better than *C*_trough_ alone (AUC, 0.645), suggesting that patients with preserved hepatic reserve may tolerate higher concentrations, whereas those with impaired reserve may develop hepatotoxicity even within conventional ranges. This concept is consistent with recent data indicating that ALBI ≥ −1.91 identifies high-risk patients even at *C*_trough_ 1–4 μg/mL (Asai et al., 2025). Therefore, risk stratification for VRC-induced hepatotoxicity should incorporate both *C*_trough_ and ALBI score rather than relying on a universal trough target.

## Limitations

The primary limitation of this study is the absence of CYP2C19 genotyping, which precluded evaluation of pharmacogenetic effects on VRC exposure and VRC-related ADRs. Second, this was a single-center study conducted in a tertiary hospital, and local population characteristics and practice patterns may limit external validity; therefore, our estimates may not be fully generalizable to other settings. Third, no systematic post-discharge follow-up was performed, and delayed-onset ADRs, particularly hepatotoxicity occurring weeks after VRC discontinuation—may have been missed. Finally, no *a priori* sample size calculation was conducted and the number of ADR events was limited, which may reduce statistical precision and increase the risk of overfitting. Larger multicenter prospective studies incorporating pharmacogenomic testing and structured post-discharge follow-up are warranted to confirm these findings across diverse patient populations.

## Conclusion

This study identified hepatotoxicity, neurological disorders, and visual symptoms as the most common ADRs associated with VRC. Most ADRs occurred within 4–7 days after treatment initiation. In addition to VRC *C*_trough_, the ALBI score demonstrated good predictive value for hepatotoxicity, with patients exhibiting an ALBI score > −2.095 at higher risk. Future multicenter, prospective studies with larger sample sizes are warranted to validate and expand upon these findings.

## Data Availability

The original contributions presented in the study are included in the article/Supplementary Material, further inquiries can be directed to the corresponding authors.
