# Supplementary material for: Voriconazole-associated adverse drug reactions in Chinese patients: incidence, clinical features and the predictive value of ALBI score for hepatotoxicity
Source: Front Pharmacol. 2026 Jul 10;17:1839608. doi: 10.3389/fphar.2026.1839608 (PMC13396025; doi:10.3389/fphar.2026.1839608)
Supplement: Supplementary file 1 [file Table1.docx]

**Table S1. Univariate and multivariate logistic analysis of risk factors for neurological disorders related to VRC**

| Variables | Univariate analysis | |
| --- | --- | --- |
|  | OR (95% CI) | *P* value |
| Female ^a^ | 2.109 (0.691-6.431) | 0.190 |
| Oral administration ^b^ | 0.429 (0.166-1.114) | 0.082 |
| VRC dose (mg/kg/day) | 1.193 (0.944-1.509) | 0.140 |
| VRC *C*_trough_ (mg/L) | 1.100 (0.879-1.376) | 0.405 |
| Age ＜60 years ^c^ | 0.756 (0.294-1.944) | 0.562 |
| CRP group ^d^  40-100 mg/L  >100 mg/L | 0.270 (0.062-1.181)  2.158 (0.856-5.438) | 0.082  0.103 |
| ALBI score | 1.549 (0.725-3.310) | 0.258 |
| Combined use of PPIs ^e^ | 1.491 (0.525-4.234) | 0.453 |
| Combined use of glucocorticoids ^f^ | 1.342 (0.438-4.110) | 0.606 |

VRC, voriconazole. *C*_trough_, trough concentration. CRP, C-reactive protein. ALBI, albumin-bilirubin. PPIs, proton pump inhibitors. OR, odds ratio. CI, confidence interval.

^a^ Compared to male.

^b^ Compared to intravenous administration.

^c^ Compared with patients aged ≥60 years.

^d^ Compared to CRP ＜40 mg/L.

^e^ Compared to without combined use of PPIs.

^f^ Compared to without combined use of glucocorticoids.

**Table S2. Univariate and multivariate logistic analysis of risk factors for visual symptoms related to VRC**

| Variables | Univariate analysis | |
| --- | --- | --- |
|  | OR (95% CI) | *P* value |
| Female ^a^ | 0.795 (0.301-2.097) | 0.642 |
| Oral administration ^b^ | 1.994 (0.449-8.851) | 0.364 |
| VRC dose (mg/kg/day) | 1.297 (1.018-1.652) | 0.035 |
| VRC *C*_trough_ (mg/L) | 0.844 (0.626-1.139) | 0.267 |
| Age ＜60 years ^c^ | 1.077 (0.375-3.090) | 0.890 |
| CRP group ^d^  40-100 mg/L  >100 mg/L | 0.495 (0.141-1.742)  0.375 (0.085-1.660) | 0.273  0.196 |
| ALBI score | 0.503 (0.190-1.333) | 0.167 |
| Combined use of PPIs ^e^ | 1.264 (0.404-3.952) | 0.687 |
| Combined use of glucocorticoids ^f^ | 0.495 (0.141-1.742) | 0.273 |

VRC, voriconazole. *C*_trough_, trough concentration. CRP, C-reactive protein. ALBI, albumin-bilirubin. PPIs, proton pump inhibitors. OR, odds ratio. CI, confidence interval.

^a^ Compared to male.

^b^ Compared to intravenous administration.

^c^ Compared with patients aged ≥60 years.

^d^ Compared to CRP ＜40 mg/L.

^e^ Compared to without combined use of PPIs.

^f^ Compared to without combined use of glucocorticoids.
